# Is Wrenn’s Strong Virtue Theory of the value of truth too strong?

**DOI:** 10.1007/s44204-025-00340-5

**Published:** 2025-10-26

**Authors:** Susanna Melkonian-Altshuler

**Affiliations:** University of Vienna, Vienna, Austria

**Keywords:** Strong Virtue Theory, Aristotelianism, Deflationism

## Abstract

In *The True and the Good*: *A Strong Virtue Theory of the Value of Truth *(2023), Wrenn argues for a strong virtue theory of the value of truth. What makes truth valuable is *Truthfulness*, a human virtue. We value truth because we ought to be Truthful, and not vice versa (state-given). Therefore, he argues that the truth of a proposition as such does not confer value to states of believing it. I disagree. I argue that Wrenn’s Strong Virtue Theory of the Value of Truth is either *too strong* or simply false. In any case, my main argument is based on a thought experiment that is supposed to challenge his theory, and pump our intuition that truth as such confers value on states of believing it (object-given). I also point to some issues that concern the compatibility of Aristotelianism and truth’s value, and also the formulation of the Strong Virtue Theory.

**Supplementary Information:**

The online version contains supplementary material available at 10.1007/s44204-025-00340-5.

## Introduction

In *The True and the Good*: *A Strong Virtue Theory of the Value of Truth* (2023), Wrenn tackles the *problem of truth’s value*. The problem is to reconcile a good theory of the nature of truth with a good theory of the value of truth. Aristotelianism about the nature of truth, for example, treats truth as a purely descriptive matter and thus struggles to explain how a purely descriptive matter could make something good to believe. Normativism about the nature of truth, on the other hand, builds value into the very nature of truth, and thus obscures the connection between what is true and how things are in the world. Wrenn’s approach to the problem of truth’s value is to adopt Aristotelianism (in particular, a propositional quantification version of deflationism) and to offer a virtue theoretic view of the value of truth, thus denying that truth as such conveys value on states of believing it.

In particular, Wrenn argues for what he calls the *Strong Virtue Theory of the Value of Truth*. In a nutshell, the theory says that truth is valuable because we ought to want/desire true beliefs and wanting/desiring true beliefs is a moral virtue – *it is to be Truthful*, where the capital ‘T’ signals that the term is technical. For Wrenn, *Truthfulness* is the character trait of valuing truth, and to value truth is to want/desire true beliefs. Thus, we get a strong virtue theory: truth is valuable because Truthfulness is a morally laudable character trait, not because truth as such is valuable. In Wrenn’s own words, there are two main components of the Strong Virtue Theory: *State Given* and *Moral*.

*State-Given* (10) says: ‘If true beliefs, as such, are valuable, they are valuable because we ought to want them, not vice versa.’ Notice the ‘ought’ in Wrenn’s preferred formulation. I will criticize it later. For now, it suffices to say that *State-Given* is inspired by Parfit’s (2001, 21; 2013) distinction between ‘object-given’ and ‘state-given’ reasons for desiring something. According to this distinction, desiring something for object-given reasons is to desire it because of its own features. For example, you might desire happiness because it’s good in itself. On the contrary, state-given reasons for desiring concern the state of desire. For example, you might desire that Harris wins the next presidential election because that desire is good—it helps you to fit in with your local community.^1^

*Moral* (9), on the other hand, says: ‘We morally ought to be Truthful. That is, we morally ought to want for what is true to be believed by ourselves and others and for what we and others believe to be true.’

While Wrenn adds *State-Given* to *Moral*, he (36) notes:

*Moral* is consistent with normativism, and it’s consistent with the value-conferral model of truth’s value.

To appreciate the value-conferral model, consider Wrenn’s own (17) formulation of it:

a proposition’s *being true* confers goodness on states of believing it (or acts of asserting it). The goodness conferred might be intrinsic, instrumental, or another sort. *Truth* is a source of goodness, and we ought to value sources of goodness.

On this model, we have object-given reasons to value truth because it’s a feature of truth that it is valuable. To defend the value-conferral model, I will proceed as follows: Putting aside issues of Aristotelianism/deflationism and normativism, I’ll present two thought experiments to pump the intuition that truth is valuable for object-given reasons and that Wrenn’s Strong Virtue Theory of the Value of Truth is either too strong or simply false. Then, I will address issues related to whether we need as a result to reject Aristotelianism/deflationism about the nature of truth. I will deny this. Finally, I will also raise some worries about how Wrenn formulates *State-Given* and *Moral*, suggesting that the conflation of the two becomes problematic: at times it seems as if Wrenn is making an argument for *Moral* instead of *State-Given*, which weakens his theory as he claims that *Moral* is independent of the Strong Virtue theory and compatible with both normativism and the value-conferral model of the value of truth.

## *Two thought experiments and aristotelianism*

As we have seen above, Wrenn argues that truth’s value derives from us human beings being virtuous. In what follows, I will argue against his Strong Virtue Theory based on two thought experiments.

### First thought experiment: against a virtue theory

Wrenn claims that Truthfulness is a moral virtue because it contributes to the overall good. This sounds as if he advocates for a *consequentialist* virtue ethics. But he also seems to advocate for an *agent-based* virtue ethics, according to which forms of normativity, including the value of happiness and the overall good, are explained by the motivational and dispositional qualities of agents. What speaks for this latter interpretation is that he says that

truth’s value derives from the moral value of *caring* about truth (p. 8, italics added), and that a good society needs members who value truth over falsehood and ignorance, for themselves and others (see back cover of the book).

This talk of *caring* about truth and *needing members who value truth over falsehood* seems to indicate that virtuous agents are at the core of Wrenn’s Strong Virtue Theory of the Value of Truth. So, if we believe that the normativity of virtue is tied to agents with the right dispositions and motivations, then suppose that we’re looking at a world where there are no human beings at all, and so being virtuous is an uninstantiated property in that world. Would Wrenn’s theory suggest that truth has *no* value in such a world? Would truth even exist? Suppose it would still be the case that grass is green, water is a liquid, and 2 and 2 are 4, etc. So, the corresponding propositions would be *truths* because they would *be true*.^2^,^3^ But if there are no human beings who could even be potentially virtuous, does it still make sense to claim that truth’s value derives from the moral virtue of *caring* about truth?

As an anonymous reviewer has pointed out, this reasoning presupposes a particular view on truthbearers, one which allows them to exist independently of people existing, and that we presumably wouldn’t be able to make the point with respect to beliefs and sentences, which are more dependent on humans. Indeed, I intend propositions to be abstract entities, and to be part of a third realm independent of thinking beings, as Frege put it (see, e.g., Frege 1956).

Wrenn, instead, might prefer beliefs because they’re more dependent on humans, and that might better fit with what he is trying to achieve, but just as I accept that my thought experiment depends on a particular view of truthbearers, Wrenn, too, has to take into account that his view of a truthbearer might just be one of many. However, note also that Wrenn himself works with propositions as the contents of beliefs (see his p. 16 or 39, for example), and so he can’t object to the fact that the thought experiments mention propositions as truthbearers.

As with respect to the reviewer’s question of whether propositions are just theoretical posits rather than accepted on the basis of intuitions, my response is that if facts are seen as true propositions, then propositions are not just theoretical posits. Frege, for example, says in ‘The Thought’ that a fact is a true thought,^4^and on the face of it, a true thought is just a true proposition. So, if a fact is a true proposition, then working with propositions, the intuitive pull of the scenario above should not be threatened. It is reasonable to assume that certain physical facts such as the fact that snow is white can exist without any humans (unlike, perhaps, social facts). And given that the thought experiments concern truth as such (in an objective sense), it would be strange to talk about human-dependent truthbearers instead.

In an attempt to respond on Wrenn’s behalf, perhaps, we could counterfactually make sense of the scenario: If there *were* people, then truth’s value could be tied to the right dispositions and motivations of agents. But this suggests that truth is not intrinsically valuable as it is dependent on people’s mental states and this is *exactly what has to be shown and thus cannot be presupposed*, so the counterfactual picture doesn’t work, at least, not initially.

If our intuition is that truth would still have value in a world where there are no people at all, then the natural answer seems to be that truth has value in such a world because truth *is valuable in itself.* If there *were* people in the world imagined, then those people’s true beliefs would have value for object-given reasons. Of course, the difficulty is to show that what exactly makes truth valuable for object-given reasons *without already presupposing that truth is valuable for object-given reasons*. But the goal of this paper is not to *prove* that truth is valuable for object-given reasons, but only to pump our intuition that this might be the case given the thought experiment presented above.

In response, one could object that, if there is any relevant intuition here concerning truth and value, it’s just that truth is valuable, and that the problem is that, upon reflection, it’s not clear what that could amount to.^5^

To respond, the intuition that I am concerned with in the thought experiments should be the same that Wrenn is concerned with, but I don’t think that the objection is fair.The point of the thought experiment is that we’re considering a case that Wrenn doesn’t, namely, a world that is void of humans. So, if Wrenn were to raise concerns about the success of the intuition pump, he would have to take the specific scenario into account. None of his arguments against the value-conferral model of truth’s value concern the specific scenario in the thought experiment. Perhaps the objector thinks that the details of the thought experiment don’t matter, as the real question simply is whether we ought to value truth (see more on this in Section 2). But even if we put things that way, we still have to consider the specifics of the presented thought experiment. If ‘we’ doesn’t simply reference the theorist’s perspective *on* a world, but humans’ perspectives *in* a world, then whether or not there are humans in a given world does matter. It’s not enough to say that it’s unclear what ‘truth is valuable’ amounts to, and that it could simply mean that we ought to value truth, as Wrenn puts it. For the intuition pump to get off the ground, we need to be more specific than that.

### Second thought experiment: against a strong virtue theory

Once more assuming that Wrenn works with an agent-based theory of virtue ethics, here is a second thought experiment: Suppose there is a world in which there *are* humans, but there are no *virtuous* humans, only *vicious* ones. Would that suggest that truth is not valuable for object-given reasons? If the answer is *no*, and truth is valuable as such, then Wrenn needs to clarify what his *Strong Virtue Theory* exactly implies: (a) the mere *potential* of virtuous beings is sufficient for the truth of *State-Given* or (b) *State-Given* requires the actual existence of virtuous humans in a given world.

If the former is the case, then there is reason for Wrenn to *weaken* his *Strong Virtue Theory*. Consider the following *Modus Ponens* argument for the value-conferral model to see why:

1. If we think truth still has value in a world where there only are vicious people, then we should adhere to the value-conferral model.
2. We do think truth still has value in a world where there are only vicious people.
3. So, we should adhere to the value conferral model.

Premise (2) is an intuition. To see the point of the argument, one has to engage with the intuition. With regard to premise (1), the idea is that the antecedent logically forces its consequent. However, Wrenn could respond that what the argument shows is that his theory is *too strong*, not that it is false. If truth has value in a world where there are only vicious people, then that is only good enough for dismissing a strong virtue theory but not a *weak virtue theory*.

**The Weak Virtue Theory of the Value of Truth**: *State-Given* doesn’t say that people actually have to be virtuous. People can also be vicious. What matters is that they have the potential to be virtuous.

But is this sort of weakening actually available to Wrenn? Recall *State-Given:*

Truth is valuable because we ought to value it, and not vice versa.

The ‘ought’ suggests that it’s our obligation to value truth, but if so, then it does matter that we *strive* to become more virtuous (see, e.g., Watson, 1997). It’s not good enough to only be potentially virtuous. Since we *ought* to be virtuous, we need to make a real effort. So, just adjusting Wrenn’s theory from strong to weak won’t undermine the consequent of premise (1).

Note also that it is not good enough to respond to (1) in the following way to defend Wrenn’s theory:

In a world populated by vicious people, truthfulness would still be a virtue assuming humans flourish under the same conditions. It just turns out that this is a world where human do not flourish.^6^

This is not good enough because the conditions of flourishing are what is at issue. If the idea is that truth as such promotes human flourishing (in claims such as ‘true beliefs lead to successful action’), then that doesn’t help Wrenn, as he is against the value-conferral model of truth’s value. Similarly, it won’t work to say that Truthfulness would still be a virtue in a vicious world because people value truth. First of all, in the given scenario, they don’t. So, Wrenn’s approach to truth’s value or even Truthfulness doesn’t hold up. This is what motivates the distinction between (a) and (b).

Second, as what is at the heart of the issue is the question of whether truth as such is valuable or not, one needs to explain why and how Truthfulness would still be a virtue in a world full of vicious people without presupposing that the conditions of flourishing would just be the same as in a world full of virtuous humans, that is, without presupposing that they would be such that people would nevertheless value true beliefs. Since this is the *explanandum*, it shouldn’t be the *explanans *(more on this in Section 2).

Perhaps yet another response to premise (1) can be provided in the context of what Wrenn says in chapter 5 about the view that truth as such confers *instrumental* value on states of believing it. His view is that truth as such does not confer instrumental value, and that it is just a psychological fact about us that we typically think of true beliefs as conducive to our ends and of false beliefs as not conducive. But that is compatible with the Strong Virtue Theory, according to Wrenn. More specifically, he says (110):

Our stereotypes aren’t driven by the instrumental goodness truth confers on belief-states. They’re driven by the facts (i) that we can’t help but choose our actions based on what we believe and (ii) that we can’t help but aim for truth in deciding what to believe. Being Truthful, though, is not just trying to get beliefs and to act on them. It involves wanting what’s true to believed, and wanting what’s believed to be true, for oneself and others. We might call truth “instrumentally valuable” in the sense that there are instrumental reasons for us to be Truthful, but that’s entirely compatible with the Strong Virtue Theory and even with a deflationary view of truth.

First, note the shift to *Truthfulness*. One might ask: Is being Truthful the same as valuing truth/saying truth has value? I will talk about this question further below when I raise concerns about the *formulation* of the Strong Virtue Theory. So, let’s put it aside for the moment. Moving forward, note that Wrenn makes a distinction between *reasons* to value something and *explaining* why we ought to value something. As we can see in the quote, he does not deny that there might be instrumental reasons to value truth (for more on this distinction see his p. 109**)**, but he does clearly deny that there is an object-given explanation of why we ought to value truth. With respect to the latter, let’s say he is right. Then, if we in turn apply the idea of *stereotypically taking truth to be end-conducive* to our thought experiment, perhaps we can say that it is compatible with a world that only has vicious people. In particular, we might be able to say that they still have the relevant stereotypes about truth and falsity even if they are vicious. So, on this interpretation then, the consequent of (1) wouldn’t follow. But it’s important to see that this is not a good defense of the Strong Virtue Theory. According to the quote above, the theory requires that one be *Truthful*, that is, that one want what’s true to be believed and what’s believed to be true, but this is inconsistent with our thought experiment as we said that they are only vicious people in the world at issue, and vicious people are *not Truthful*. It would be good for the vicious people to *want* true beliefs, but as they are vicious, they are not Truthful in that sense. If they are vicious, then their desires cannot confer instrumental value onto their beliefs. Therefore, Wrenn’s response to the instrumental value version of the value-conferral model does not work in the context of our thought experiment, and the consequent in (1) is not undermined. What will make the difference to the instrumental goodness of believing true beliefs in the world described is a proposition’s *being* true. Or alternatively, people need to actually be Truthful, and *act* on their stereotypes if we want a state-given explanation.^7^ Either way, it seems that the given defense is undermined. Given space limitations, I’ll leave it for another day to decide whether his responses in the other chapters regarding the intrinsic version and the epistemic version of the value-conferral model work. Moving on, I will briefly address the issue of the *apparent incompatibilism* between the value-conferral model of the value of truth and Aristotelianism/deflationism about the nature of truth.

### Aristotelianism and truth’s value

Above, I put some pressure on Wrenn’s claim that truth doesn’t have value for object-given reasons. Supposing the value-conferral model is correct, should we give up *Aristotelianism*? According to Wrenn, Aristotelianism says that truth is a matter of whether things are as they are said/thought to be. ‘True’ does not pick out a normative property. But does this suggest that Aristotelianism is incompatible with the value-conferral model? An affirmative answer presupposes that the value-conferral model requires a kind of normativism about truth, and since Aristotelianism is incompatible with normativism, the model is incompatible with Aristotelianism.

But I think we need to pause here. I believe that there are ways of avoiding the outcome that Aristotelianism is incompatible with the value-conferral model *even if the latter requires normativism about truth*. Perhaps we can appreciate this if we ask the following question: Does a certain theory of the nature of truth *have* to be compatible with a certain theory of the value of truth? So, does any view on the value of truth *have* to be compatible with Aristotelianism?

Isn’t the one asking what *truth* is, and the other what the *value* of truth is? This is something that Wrenn himself notes in the context of describing why Aristotelianism doesn’t deal with the problems that normativism deals with. He says (87):

Aristotelianism doesn’t face the problems described here. For Aristotelians, the question of a proposition’s truth is the question whether things are as it says they are. The proposition’s fitness for belief is a separate question. There is nothing normative built into *truth* or TRUE, and so truth-attributions have no built-in attribution of fitness to believe. Aristotelians can accept that it’s ordinarily wrong to believe (or assert) what isn’t true. They can also accept that it’s ordinarily right to believe (or assert) what is true. Aristotelians just deny that those rules are part of the very meaning of ‘true’, the content of TRUE, or the nature of *truth.* Truth, from the Aristotelian point of view, might be normatively relevant, but it’s not normative.

I agree with this quote in the sense that questions about the nature of truth and questions about the value of truth are separate questions. There is still an open question of whether the thought experiments presented for truth’s value for object-given reasons imply that truth is *normative*, and not just *normatively relevant*. I’d like to leave this open. The thought experiments might give us reasons to believe that truth is normative. But it’s not clear that Aristotelians have a problem. As Wrenn himself points out, there are at least two forms of normativism. According to Wrenn (71), *metaphysical normativism* is the view that truth is a normative property. Truth as such is good. *Conceptual normativism* is the view that TRUTH is a normative concept. It’s part of the content of TRUTH that true propositions are good to believe. And we can add that if Aristotelianism is incompatible with one of these forms that doesn’t necessarily mean that it is also incompatible with the other. Just in the same way that many are minimalists about the concept of truth but not the property of truth (e.g., Wright, 1992). According to Wright, there is a plural truth *property* and truth is normative, although the *concept* of truth is minimal, that is, it is accounted for by both the disquotational schema and the operator schema, '‘p’ is true if, and only if, p', and ‘it is true that p if, and only if, p’, plus some other platitudes about truth (see his 1992, 34–5). So perhaps, in the case of the value of truth, truth as such is valuable in the sense that the *concept* of truth is normative, while the *property* of truth is not (or vice versa). This is something to investigate further.^8^ Last but not least, there might be *independent* reasons for avoiding Aristotelianism. Wrenn argues against normativism by discussing two kinds of propositions: *blindspots* and *brightspots*. Blindspots are propositions that could be true, but couldn’t be truly believed**.** To use Wrenn’s example: <It’s raining, and no one believes it’s raining.> is a blindspot proposition. The problem for normativism is that blindspots can’t be fit to believe, as they can’t be truly believed, although they can be true. Brightspots, on the other hand, are propositions that could be false, but couldn’t be falsely believed. Wrenn’s example is: <Seven is a prime number, and someone believes seven is prime.> Brightspots can’t be unfit to believe, as they can’t be falsely believed, although they can be false. Thus, the takeaway is that truth as such is not valuable, and falsehood as such is not disvaluable. But even if we suppose that normativism is wrong, it’s not clear that that shows that Aristotelianism is correct. In chapter 4, Wrenn rigorously argues why we should not embrace normativism, but he has not shown that it’s being false *entails Aristotelianism’s being correct*. For all we know, Aristotelianism, too, could be wrong. So, what I am missing in the book is why Aristotelianism as such is a good theory. On p. 70, so approximately halfway through the book, Wrenn says:

I haven’t [yet] argued for *State-Given* beyond pointing out that Aristotelians (and especially deflationists) should find it attractive. But why be an Aristotelian? Why not avoid the problem by embracing Normativism?

Wrenn makes negative arguments for why not to embrace normativism in the sense that he either argues that normativism is wrong or that views that combine descriptive and normative elements as part of the concept of truth (making it a thick concept) are wrong (see chapter 4). But he does not motivate Aristotelianism in its own right, and one might find that to be required for fully embracing Aristotelianism.

## Problems with the Formulation of the Strong Virtue theory of the value of truth

Throughout the book, I see various problems with the way in which the Strong Virtue Theory is formulated. So, here are some things to keep in mind about Wrenn’s views when reading the next sections:

- 1) to value truth is to want true beliefs,
- 2) to want true beliefs is to be Truthful,
- hence,
- 3) to value truth is to be Truthful.

In what follows, I will discuss some of the consequences of 1)–3) in the context of *State-Given* and *Moral*.

### First problem: *moral*’s endorsement of *state-given*

The first problem is that *Moral* and *State-Given* are not independent of each other. Here is *State-Given* again:

*State-Given Truth* is not a property of propositions that confers value on states of believing them; if we ought **to value truth**, it isn’t because *valuing truth* is a fitting response to the goodness conferred on beliefs by the truth of their contents. If true beliefs, as such, are valuable, they are valuable because we ought to want them, not vice versa. (10)

*Moral* again:

*Moral* We morally ought to be Truthful. That is, we morally ought to want for what is true to be believed by ourselves and others and for what we and others believe to be true. (p. 9)

Here is how he defines *Truthfulness*:

I’ll use ‘Truthfulness’ to name the *trait* of valuing truth. People who value truth are ‘Truthful’ (25).

As we can see, in *State-Given*, Wrenn says that if we ought to value truth, then so and so, and in *Moral* he says we morally ought to be Truthful. But why is *Moral* basically confirming *State-Given*’s content as morally correct? *State-Given* says that the explanation of why truth is valuable is that we ought to want true beliefs, and *Moral* says that we *morally* ought to want true beliefs, etc. So, it turns out that *Moral* is supporting *State-Given* as it says to want true beliefs is a moral virtue. Is that a problem? Even valuing truth itself is defined via *wants*. Wrenn’s says (68):

[*Value*:] Valuing truth is wanting what’s true to be believed and wanting what’s believed to be true.

Now, Wrenn claims that *Moral* is consistent with the value-conferral model (36):

*Moral* is consistent with normativism, and it’s consistent with the value-conferral model of truth’s value. The Strong Virtue Theory goes further. It adds *State-Given* to *Moral*, denying that the truth of a proposition confers value on states of believing it.

If *Moral* is consistent with the value-conferral model, then shouldn’t *Moral* be neutral towards *State-Given*? When Wrenn explains the concept of *state-given* more generally, making use of Parfit’s (2001, 21; 2013) work, there is no ‘ought’ built into the idea. Wrenn says (37, italics added):

State-given reasons for desiring concern the *state* of desire, rather than its object. I might want the local football team to win because that *desire* helps me fit in with my neighbours. If I do, the reasons for my desire are ‘state-given’. They flow from the *state* of desiring something, rather than from the thing desired.

One problem with this formulation is that it misrepresents Parfit’s notion of state-given reasons. These are reasons deriving from the *value* of the state, not from having the state itself.^9^ Putting that aside, consider the following reformulation of Wrenn’s *State-Given* without the final ‘ought’:

*State-Given-Revised:* *Truth* is not a property of propositions that confers value on states of believing them; if we ought to value truth, it isn’t because *valuing truth* is a fitting response to the goodness conferred on beliefs by the truth of their contents. If true beliefs, as such, are valuable, they are valuable because we want them, not vice versa.

For those who want a state-given explanation, this seems to be a more correct version of *State-Given*. The question is why Wrenn doesn’t consider it or formulate it. Perhaps because state-given reasons as such are not good enough to validate why we ought to value truth/be Truthful, as we can’t get an *ought* from an *is*. An *ought*, on the other hand, makes better sense of why we should value truth/be Truthful. But it seems that this should not matter, unless *Moral* as such is the *explanandum*. But is it? Isn’t *truth’s value* the *explanandum*? Indeed, it seems as if Wrenn is equating these things, as he says **(**69**):**

even if *Moral* is true, there might be more to the value of truth than the virtue of Truthfulness. The next few chapters make the case for Aristotelianism and *State-Given*, the other, more controversial, half of the Strong Virtue Theory.

Aren’t we trying to explain and give a theory of the value of truth? And isn’t it a different question whether or not we *ought* to value truth/be Truthful?^10^ Perrine (2024) makes a similar point. He points out that reasons for why we ought to be Truthful aren’t necessarily reasons for why truth is valuable. Considering that, according to Wrenn, to be Truthful is to desire true beliefs, Perrine argues that ‘it is generally false that if there are reasons that explain why we ought to desire φ, then φ is valuable’ (ibid, 09.9.). He considers an example by Crisp (2000, 459): there is a reason that explains why one ought to desire a saucer of mud. The reason is that if one doesn’t, an evil demon will torment them. But it does not follow that the saucer of mud is valuable.

### Second problem: the equation of *valuing* and* desiring*

Perhaps, our task is first to explain what the value of truth is, before we explain why we ought to value truth/be Truthful. Indeed, Wrenn seems to agree that we actually have object-given reasons for *Moral* which suggests that we need to distinguish between why truth is valuable and why we ought to value truth/be Truthful. He says (35):

*Moral* employs the ‘ought’ of character. Truthful people are motivated by truth. They seek out new truths for themselves and others. They try to identify and overcome their erroneous beliefs, in themselves and others. According to *Moral*, we ought to be such people.

This says that Truthful people are motivated by *truth*, so how could we say that if we disregard the value conferral model? If Truthfulness is a feature of ours, it’s the *trait* of *valuing* truth; then valuing truth suggests valuing something other than a feature of ours, unless we think that truth is subjective, but given that Wrenn works with Aristotelianism and deflationism, he doesn’t seem to think that. So, truth’s value/why truth is valuable should not be equated with *Truthfulness*. But as we have seen, in *Moral* Wrenn (implicitly) defines being Truthful as wanting what is believed to be true and what is true to be believed, and there is also evidence that he defines *truth’s value *via* desires*. When Wrenn describes Horwich’s proposal (which is irrelevant for us), he says that he agrees with Horwich that the problem of explaining truth’s value comes down to ‘explaining its desirability and its correctness’ (51). And later, since he agrees with Horwich, he conjoins the criteria of desirability and correctness ‘into a more general statement of the idea that truth is valuable’ (53):

*Valuable* One ought to want to believe what is true, and only what is true.

Desiring truth is a good thing and important, but I don’t see why truth’s being desirable is the same as truth’s being valuable. One could also value truth in virtue of acting in accordance with truth or in virtue of simply having true beliefs. Perhaps someone who values truth *also desires truth*. But *prima facie* it is not clear why the act of valuing truth must suggest anything about our desires (see, e.g., Smith, 1994 on this). I can value truth through *doing the right things*. Does this mean I value truth in Wrenn’s sense? Perhaps one who does the right thing values truth in Wrenn’s sense, but then what matters is what we do, not what our state of minds are, i.e., whether we want true beliefs, etc. Perhaps in defense of Wrenn (and Horwich), we can say that the formulation in *Valuable* is definitional. It is a consequence of *State-Given*: for truth to be valuable is for one to want things in the sense of Wrenn’s theory*.* Still, the definition undermines his project of wanting to *explain* truth’s value in terms of *State-Given*, where the principle at issue is supposed to ground/explain truth’s value and not be a kind of *definiens* that could easily be used instead of the *definiendum*, which makes the *definiens* an *explanandum* instead of an *explanans*. Compare with ‘unmarried adult man’ for ‘bachelor’. When we substitute the one for the other, we get a definition, but it’s not like we have a fruitful explanation of why someone is a bachelor if we say because they are an unmarried adult man. Even if the explanation is *analytically correct*, we usually want something more substantial—such as he is a bachelor because he works too much, or because he is nerdy, or because he is shy, etc.

### Third problem: a conflation of *explanandum* and *explanans*

Another example of the conflation of *explanandum* and *explanans* seems to be the following claim:

*[Virtue]* On the Strong Virtue Theory, we ought to value truth because valuing truth is virtuous (11)

Here, we seem to explain *why we ought to value truth*. And it seems that this was also the goal of *State-Given* (falsely). Again, Wrenn’s *State-Given* says:

*Truth* is not a property of propositions that confers value on states of believing them; if **we ought to value truth**, it isn’t because *valuing truth* is a fitting response to the goodness conferred on beliefs by the truth of their contents. If true beliefs, as such, are valuable, they are valuable because we ought to want them, not vice versa. (10)

However, since, in *Value*, we saw that, for Wrenn, to value truth is to want truth beliefs, it seems as if *Virtue* is delivering an explanation of why we ought to *want true beliefs*. But if so, then Wrenn’s explanation is this: We ought to want true beliefs because wanting true beliefs is virtuous. In other words, *State-Given* now provides the *explanandum*, not the *explanans*. ‘To ought to want true beliefs’ is both the *explanans* and the *explanandum* in Wrenn’s theory. But in a good explanation, the *explanans* is something other than the *explanandum*, something that grounds the *explanandum*, so we need a different *explanans;* perhaps we need *object-given reasons*: we ought to want true beliefs because truth is valuable as such, where we are not simply equating why we ought to want true beliefs with why truth is valuable.

However, maybe all this is uncharitable. An alternative view is that *Virtue* explains *State-Given*. First, *State-Given* (falsely) explains truth’s value in terms of ‘we ought to want truth beliefs’, and *Virtue*, in turn, explains why we ought to want true beliefs (as to value truth is to want true beliefs). But if this is the case, then *Virtue* also seems to explain why we ought to be Truthful as we have seen previously that, for Wrenn, ‘valuing truth’ and ‘being Truthful’ are coextensive. We saw this when we compared *State-Given* with *Moral*, and the same can be observed when we look at Wrenn’s formulation of the value conferral model (17):

It says a proposition’s *being true* confers goodness on states of believing it (or acts of asserting it). The goodness conferred might be intrinsic, instrumental, or another sort. *Truth* is a source of goodness, and we ought to value sources of goodness. So, **we ought to value truth. We ought to be Truthful.**

Here it seems (again) as if to value truth *is just to be Truthful/just is to be virtuous*. If this interpretation is correct, then we seem to say the following:

*Virtue Revised* On the Strong Virtue Theory, we ought to be Truthful because being Truthful is virtuous.

Now, we seem to be going second-order: we’re saying that we ought to be Truthful because *Truthfulness gets its value through other virtues*. Suppose it’s an instrumental virtue, not an intrinsic virtue. Perhaps this is not what Wrenn wants to imply as one is supposed to be moral/virtuous/Truthful on his theory because it leads to the overall good (see, e.g., p. 19), not because being Truthful has instrumental value. Finally, there is also the possibility of understanding the idea of Truthfulness’ being virtuous as implying that we *ought to value Truthfulness*:

*Valuing Truthfulness* We ought to be Truthful because we ought to value Truthfulness.

Here, we don’t seem to say anything new. But this reading, then, does get us what Wrenn wants as long as we replace the *explanandum* with ‘we ought to value truth’, so then it’s the value of Truthfulness that gets us to value truth with the caveat that now we need to first explain why we ought to value Truthfulness. If the explanation is that it’s virtuous to do so, then we seem to be back to square one.

## Supplementary Information

Below is the link to the electronic supplementary material.

ESM1 (DOCX 25.7 KB)

## Data Availability

No data was generated or analyzed.

## Footnotes

1 Parfit denies that there are state-given reasons. According to him, there are only object-given reasons.

2 We could also imagine a scenario, according to which, the propositions at issue in the imagined world don’t have truth, and thus are not truths, even though they are true in *our* world. But that’s not our thought experiment here. Thanks to Steve Finlay for drawing my attention to this.

3 We’re also putting aside the view that without language and minds, there are no propositions (see, e.g., King, 2007).

4 Note that a *thought *for Frege is the objective, timeless, and eternal sense of a complete declarative sentence, and not the creation of the thinking person (for more on this, see Frege 1956).

5 Thanks to an anonymous reviewer for this interesting response.

6 Thanks to an anonymous reviewer for this interesting response.

7 Note that, according to Wrenn, people could also be Truthful for object-given reasons. This becomes clear when he says: (89): *Moral* assures us that Truthfulness is a virtue, but *State-Given* says that’s not because of the goodness *truth* confers on beliefs, for *truth* confers no such goodness. These lines imply that people actually having to be Truthful does not force a state-given explanation, and an object-given explanation can be provided instead. So, this is another way in which the defense presented above is undermined.

8 To be fair to Wrenn, he does say that there could be a kind of *deflationary normativism/normative deflationism* (see his p. 83). Price (2003), for one, has proposed such a view, according to which, truth doesn’t have to be substantive to be normative. Unfortunately, I don’t have the space to investigate its merit in this paper.

9 I thank Steve Finlay for this comment.

10 Some deny that these are different questions; see, e.g., Scanlon’s (1998) buck-passer argument. For an objection to buck-passing, see, e.g., Rabinowicz and Ronnow-Rasmussen (2004).
