# Supplementary material for: Is Wrenn’s Strong Virtue Theory of the value of truth too strong?
Source: Asian J Philos. 2025 Oct 26;4(2):118. doi: 10.1007/s44204-025-00340-5 (PMC12553566; doi:10.1007/s44204-025-00340-5)
Supplement: Supplementary file 1 — (DOCX 25.7 KB) [file 44204_2025_340_MOESM1_ESM.docx]

**Responses to Reviewer 1**

I thank Reviewer 1 for reading my article and providing interesting comments. Below I quote each of the comments and respond to them. I also indicate where I’ve made changes in response to the comments.

**1. Comment: “**In section 1.1 (pg. 3), the author begins developing an intuition pump meant to motivate the idea that truth is valuable to some features of truth itself, even if which features remains unclear.”

**Response:** Since the idea is that truth is valuable in itself, the phrase ‘valuable to some **features** of truth itself’ seems misleading. The idea is that a true belief, for example, is valuable because *it is true*. Truth itself is the relevant feature. If we start identifying other features, **alleged** features of truth (such as truth is the goal of inquiry or truth leads to successful actions etc.), then we lose our focus on the issue at hand. But perhaps the reviewer could clarify what they have in mind if I misunderstood them.

**2. Comment**: “The author invites to consider a world devoid of human beings, and wonder whether truth would be valuable in such a world. Along the way, the author observes that ‘So, the propositions at issue would be truths because they would be true.’ Notice, though, that this point presupposes a particular view on truth-bearers, one which allows them to exist independently of people existing. There are plenty of truth-bearers (such as beliefs or sentences) which would result in there being no truths in such a world.”

**Response:** Yes, thanks for the great observation. I intended truthbearers to be like Fregean propositions, which are abstract entities, and part of a third realm. I have added now that we presumably wouldn’t be able to make the same point with respect to beliefs and sentences, and that the thought experiment depends on a particular view of truthbearers, **see page 4, please**.

**3. Comment:** “Wrenn appears to prefer beliefs, for example. To be fair, the author mentions this latter scenario in a footnote (where there are no truths), but it's unclear he can just leave it aside so easily. After all, assuming that propositions are truth-bears might be problematic because propositions are plausibly accepted as theoretical posits (to explain a variety of phenomena) rather than accepted on the basis of intuitions. If this right, it threatens to the intuitive pull of the scenario devised by the author.”

**Response:** Just as I now accept that my thought experiment is dependent on a particular view of truthbearers, Wrenn, too, has to take into account that his view of a truthbearer might just be one of many. Wrenn might prefer beliefs because they’re more dependent on humans, and so they might fit better with what he is trying to achieve, in the same way what I am trying to do with the thought experiment works better with truthbearers that are not dependent on humans.

Besides, Wrenn himself works with propositions as the contents of beliefs (see his p. 16 or 39, for example), and so he can’t object to the fact that the thought experiments mention propositions as truthbearers. Given that the thought experiments concern truth as such, it would be strange to talk about human-dependent beliefs as truthbearers instead.

As with respect to whether propositions are just theoretical posits or not, if *facts are seen as true propositions* (see, *facts as truths* view), then propositions are not just theoretical posits, as certain facts such as the fact that snow is white can exist without any humans, unlike, perhaps, social facts. So, I have now made explicit that we could have a view of facts as truths and so true propositions would be as real as facts. Frege, for example, says in ‘The Thought’ that a fact is a true thought, and on the face of it a true thought is just a true proposition. So, a fact is a true proposition. I hope that it makes the scenario more intuitive, **see page 4, please**. Thanks for the comment!

**4. Comment:** “Leaving aside this criticism, we could imagine Wrenn pointing out that, if there is any relevant intuition here concerning truth and value, it's just that truth is valuable. The problem is that, upon reflection, it's not clear what that could amount to.”

**Response:** Thanks for the comment. It’s the very idea that there are no humans that is supposed to pump the intuition that truth is not valuable in the sense that it’s valuable because *humans value it*. Perhaps the reviewer thinks that that doesn’t matter as the real question is whether truth is a virtue in such a world where there are no humans/whether we still ought to value truth in such a world. But even if we bring in ‘ought’ it seems to me that if there are no humans, then it seems awkward to say we *ought to value truth in such a world*, where ‘we’ doesn’t simply reference the theorist’s perspective *on* a world, but humans’ perspectives *in* a world, and the latter doesn’t make sense given the thought experiment of there being no humans at all. I’ve now added this to the discussion to make my point clearer, **please see page 5**.

**5. Comment:** “Indeed, Wrenn devotes several chapters to arguing that various ways of cashing out this intuition (including truth having instrumental value) do not hold up. So, we could imagine Wrenn raising concerns about the success of the intuition pump itself. Is that initial intuition as finely articulated as the author assumes? (And this response from Wrenn, along with how he argues against various accounts of truth's value, surely should affect the counterfactual defense offered on Wrenn's behalf, starting on page 5, line 11.)”

**Response: Please see p. 5.** The intuition that I am concerned with in the thought experiments should be the same that Wrenn is concerned with, but the point of the thought experiment is that we’re considering a case that Wrenn *doesn’t*, namely, a world that is void of humans. So, if Wrenn were to raise concerns about the success of the intuition pump he would have to take the specific scenario into account. None of his arguments against the value-conferral model of truth’s value concern the specific scenario in the thought experiment. (As for the counterfactual defense offered on Wrenn’s behalf, starting on page 5, line 11, I am assuming that the reviewer is referring here mainly to what they discuss in the next comment, so I will address their point below.)

**6. Comment:** “In section 1.2 (on page 5), the author invites us to consider a world where everyone is vicious, which includes their being Untruthful. Admittedly, I find the author's take on this scenario a bit puzzling. The author asks, ‘Would that suggest that truth is not valuable for object-given reasons?’ And the author follows us considering what is advanced as a natural answer: ‘If the answer is no, and truth is valuable as such, then Wrenn needs to clarify what his Strong Virtue Theory exactly implies: (a) the mere potential of virtuous beings is sufficient for the truth of State-Given or (b) State-Given requires the actual existence of virtuous humans in a given world.’ I find this puzzling because there seems to be a much more natural answer to offer on Wrenn's behalf. In a world populated by vicious people, assuming humans flourish under the same conditions, Truthfulness would still be a virtue. It just turns out that this is a world where human do not flourish.”

**Response**: **Please see page 7.** It seems that we need to spell out what ‘under the same conditions’ means. First of all, what conditions, exactly? If the idea is that truths as such promotes human flourishing in the sense, say, that truth beliefs lead to successful actions, then that doesn’t help Wrenn, obviously, as he is against the value-conferral model of truth’s value. So, one needs to explain why and how Truthfulness would still be a virtue in a world full of vicious people without implying that Truthfulness is a virtue in such a world because *people value true beliefs*. Since this is the *explanandum*, it shouldn’t be the *explanans*. Plus, in the world described people don’t value true beliefs. So, Wrenn’s approach to truth’s value or even Truthfulness doesn’t hold up. We can’t say that truthfulness would be a virtue because people value truth. They clearly don’t. This is what motivates the distinction between (a) and (b). I’ve now added this response to the relevant passages. I hope the additions help making my take on the described scenario less puzzling.

**7. Comment:** “It’s difficult to evaluate section 1.3 of the article, given many points made call back to these points made in sections 1.1. and 1.2. It's clear, though, that adjustments made to 1.1. and 1.2 will surely require also making compensatory adjustments in 1.3.”

**Response**: Thanks for the comment. 1.3. is mainly concerned with Aristotelianism as such (and whether it is compatible with the value-conferral model). However, I start the section with **supposing** that the value-conferral model is correct, so the idea is not that *it is* correct. So, it’s not clear how much turns on section 1.2., and 1.1. It is true that I also consider whether the thought experiments imply Normativism, but I also say that I’ve to leave the question open. As in the first draft, I say:

There is still an open question of whether the thought experiments presented for truth’s value for object-given reasons imply that truth is *normative*, and not just *normatively relevant*. I’d like to leave this open. The thought experiments might give us reasons to believe that truth is normative. But it’s not clear that Aristotelians have a problem. As Wrenn himself points out, there are at least two forms of normativism […].

I realize that I have not considered all of Wrenn’s arguments and objections, but given that potential worlds are never taken into account in Wrenn’s arguments and objections against the value-conferral model, I believe that the discussed thought experiments are still relevant. And I hope the reviewer sees it the same way.
